# Changes in paranasal sinus mucosa due to the nasopharyngian obstruction

**DOI:** 10.1186/2045-7022-3-S2-P16

**Published:** 2013-07-16

**Authors:** Felicia Manole

**Affiliations:** 1 Faculty of Medicine Oradea, Oradea, Romania; 2 Faculty of Medicine Oradea, ENT, Oradea, Romania

## Background

The purpose of the study is to determine the effects of the artificially induced mechanical obstruction on the physiopathology of the paranasal sinus mucosa, taking into consideration an experimental model on animals (rabbit).

## Methods

The study was performed on 20 New Zealand white rabbits, distributed into two equal groups. In the case of the first group of 10 experimental animals, we induced the model of ipsilateral double obstruction, the opposite side being left intact as term of comparison. Thus we produced the mechanical obstruction of the nasopharynx at the level of the choanal orifices as well as the obstruction at the level of ostiomeatal complex. In the case of the second experimental group we induced the model of the double counterlateral obstruction: the obstruction of the nasopharynx at the level of the choanal orifice and the counterlateral obstruction of the ostiomeatal complex. We studied and determined the qualitative changes of the pH and the microbiology of the paranasal sinus mucosa.

## Results

the study determines: 1. pH statistical average and standard deviations of the maxillary sinus mucosa.2. The isolated germs was: Staphilococcus aureus, Streptococcus pneumoniae, Haemophilus influenzae. Multigerm association was present in the case of the double ipsilateral obstruction.3. The reversed correlation between the pH values and the presence of multiple germs in the sinus mucosa.

## Conclusions

The experimentally induced obstruction of the nasopharynx, modifies the ventilation of the nasal fossae and leads to morphophysiological changes in sinus mucosa, causing inflammation. As compared to the normal values, the pH average values of the maxillary sinus mucosa in the obstructed side are significantly lower (p<0.001). It is noticeable that there are no significant statistic differences between maxillary sinus mucosa pH values in the double ipsilateral obstruction and the obstruction of the ostiomeatal complex (p>0.05), thus certifying that the nasopharynx contributes indirectly to the ventilation of the paranasal sinuses.

